# Rurality as a Risk Factor for Attempted Suicide and Death by Suicide in Ontario, Canada

**DOI:** 10.1177/07067437211053300

**Published:** 2021-11-18

**Authors:** Rebecca Barry, Jürgen Rehm, Claire de Oliveira, Peter Gozdyra, Simon Chen, Paul Kurdyak

**Affiliations:** 1 7938 University of Toronto, Toronto, Ontario; 2 7978 Centre for Addiction and Mental Health, Toronto, Ontario, Canada; 3 9169 Dresden University of Technology, Dresden, Germany; 4 Centre for Health Economics and Hull York Medical School, 8748 University of York, York, United Kingdom; 5 ICES, Toronto, Ontario, Canada

**Keywords:** rurality, suicide, suicide attempts

## Abstract

**Objective:**

This study aims to examine rural and urban differences in attempted suicide and death by suicide in Ontario, Canada.

**Method:**

This is a population-based nested case-control study. Data were obtained from administrative databases held at ICES, which capture all hospital and emergency department visits across Ontario between 2007 and 2017. All adults living in Ontario who attempted suicide or died by suicide are included in the study, and controls were matched by sex and age. Suicides were captured using vital statistics. Suicide attempts were determined using emergency department service codes.

**Results:**

Rurality is a risk factor for attempted suicide and death by suicide. Rural males are more likely to die by suicide compared with urban males (adjusted odds ratio(AOR) = 1.70, 95% confidence interval (CI), 1.49 to 1.95), and the odds of death by suicide increase with increasing levels of rurality. Rural males and females have an increased risk of attempted suicide compared with their urban counterparts (males: AOR = 1.37, 95% CI, 1.24 to 1.50) (females: AOR = 1.26, 95% CI,  1.14 to 1.39), with a pattern of increasing risk of suicide attempts with increasing rurality. Rural females are not at increased risk of suicide compared with urban females (AOR = 1.08, 95% CI, 0.80 to 1.45). Sensitivity analyses corroborated the results.

**Conclusions:**

Rural males are almost two times more likely to die by suicide compared with urban males, and both rural males and females have an elevated risk of suicide attempts compared with urban residents. Future research should examine potential mediators of the relationship between rurality and suicide.

## Abrégé

**Objectif:**

La présente étude vise à examiner les différences rurales et urbaines des tentatives de suicide et des décès par suicide en Ontario, Canada.

**Méthode:**

Il s’agit d’une étude cas-témoins emboîtés dans la population. Les données ont été obtenues des bases de données administratives tenues à l’ICES, qui contiennent toutes les visites à l’hôpital et au service d’urgence en Ontario entre 2007 et 2017. Tous les adultes habitant l’Ontario qui ont tenté de se suicider ou qui sont décédés par suicide sont inclus dans l’étude et les témoins ont été appariés par sexe et par âge. Les suicides ont été repérés à l’aide des statistiques vitales. Les tentatives de suicide ont été déterminées à l’aide des codes de service du service d’urgence.

**Résultats:**

La ruralité est un facteur de risque des tentatives de suicide et des décès par suicide. Les hommes en milieu rural sont plus susceptibles de mourir par suicide comparés aux homes en milieu urbain (Rapport de Cotes Ajustées (RCA) = 1,70; IC à 95%1,49 à 1,95), et les probabilités de décès par suicide augmentent avec les niveaux croissants de ruralité. Les hommes et les femmes en milieu rural ont un risque accru de tentatives de suicide comparativement à leurs homonymes en milieu urbain (hommes : RCA = 1,37; IC à 95% 1,24 à 1,50); (femmes : RCA = 1,26; IC à 95% 1,14 à 1,39) et ont un modèle de risque croissant de tentatives de suicide avec la ruralité croissante. Les femmes en milieu rural ne sont pas à risque accru de suicide comparativement aux femmes en milieu urbain (RCA = 1,08; IC à 95% 0,80 à 1,45). Les analyses de sensibilité ont corroboré les résultats.

**Conclusions:**

Les hommes en milieu rural sont presque deux fois plus susceptibles de mourir par suicide comparativement aux hommes en milieu urbain, et les hommes comme les femmes en milieu rural ont un risque élevé de tentatives de suicide comparé aux résidents urbains. La future recherche devrait examiner les médiateurs potentiels de la relation entre ruralité et suicide.

## Introduction

In Canada, suicide is the second leading cause of death amongst 15–34-year-olds, and the ninth leading cause of death amongst all Canadians.^1^ Additionally, for every death by suicide, there are an estimated 25 suicide attempts.^2^ People living in more rural areas have been found to have a higher risk of suicide than those living in more urban areas.^3^ The association between rurality and increased risk of suicide has been indicated in the United States, China, and Australia, amongst other countries.^3,4^ However, there is conflicting evidence from Canada regarding the relationship between rurality and suicide, with some studies showing an association, while others do not show such an association.^3,5,6^ The most recent study to compare risk of suicide by rural–urban status in any jurisdiction in Canada uses data from 1991 to 2001, and this study did not show an association between rurality and suicide.^5^ There are more studies in the United States that examine rurality as a risk factor for suicide; however, none have used a population-based cohort to examine the association focally, and most use an ecological study design (e.g., pooled data at the county level), which is prone to bias.^3^ Ecological study designs are prone to ecological fallacy, because associations found at the aggregate group level cannot be assumed to apply at the individual level.^7,8^

Moreover, few studies have examined differences between urban and rural suicide attempts. All studies from the United States since 2006 have relied on smaller surveys (<20,000 people) and self-reported outcomes for suicide attempts, and results have been mixed.^3^ In Canada, the only published literature that reports rural versus urban suicide attempts are studies examining senior home care recipients, and youth ages 12–17, which limits the generalizability of the studies’ findings.^9,10^ Nonetheless, both of these studies indicate that those living in rural areas may be at increased risk. This study is important because there is evidence suggesting that disparities in suicide and suicide attempts between rural and urban residents may be increasing over time^11,12^ The objective of this study was to examine rural–urban suicide and suicide attempts using a population-based cohort to determine whether disparities exist.

## Methods

### Study Design

This study uses a nested matched case-control design. Cases were defined as all adults (ages 18 and over) living in Ontario who died by suicide between April 1, 2007 and December 31, 2015. Controls were age- and sex-matched individuals who were alive at the time of the matched case's death by suicide. Four controls were matched for every suicide case. For attempted suicide, cases were defined as all adults who attempted suicide between April 1, 2007 and March 31, 2016. We only used the first suicide attempt. We defined the first suicide attempt as the first suicide attempt observed within the cohort, with a 1-year lookback window. Controls were age- and sex-matched and must not have previously attempted suicide at the time of the matched case's suicide attempt. Two controls were matched to every suicide attempt case. The index date is the date of the suicide attempt or death by suicide.

### Data Sources

Data include administrative databases held at ICES in Toronto, Ontario. ICES is an independent, nonprofit research institute whose legal status under Ontario's health information privacy law allows it to collect and analyze health care and demographic data, without consent, for health system evaluation and improvement. These databases include the following: the Registered Person Databasefiles, which includes individual's age and sex and eligibility for public health care insurance; Vital Statistics Death (Office of the Registrar General – Deaths), which provide information on date and cause of death, including suicide-related deaths; the Canadian Institute for Health Information National Ambulatory Care Reporting System, which includes information on all Emergency Department visits, including suicide attempts; census data, which provide information on neighbourhood-level income quintile, metropolitan influence zone (MIZ), and degree of rurality of residence; the Ontario marginalization index for residential instability and dependency; and the Immigration, Refugees and Citizenship Canada's Permanent Resident Database, which provide information on individuals’ migrant status. The Vital Statistics cause of death field was found to have over 95% sensitivity when compared with coroner-confirmed suicides,^13^ and the algorithm for suicide attempts has also been validated.^14^ These datasets were linked using unique encoded identifiers and analyzed at ICES.

### Outcomes: Death by Suicide and Attempted Suicide

Suicide is defined using service codes delivered in the emergency department (E950-E959 cause of death (COD) or X60-X84, Y10-Y19, Y28) and validated using coroner's data.^13^ Suicide attempts is based on emergency department presentation with self-harm codes X60-X84, Y10-Y19, and Y28.^14^

### Exposure: Rurality

We used the Rurality Index of Ontario (RIO) score developed by the Ontario Ministry of Health and Long-Term Care and the Ontario Medical Association.^15,16^ Each community has a score; scores are assigned to individual study participants using postal codes. Scores range from 0 to 100, with scores from 0 to 9 considered as urban, scores from 10 to 44 considered small town, and scores greater than 44 considered rural.^15,16^ Although the RIO is continuous, a one-point increase in rurality is not easily translated into policy changes, and a categorical classification is more intuitive. RIO scores are based on community population and density, travel time to nearest basic referral centre, and travel time to nearest advanced referral centre.^15,16^

We used a second measure of rurality as a sensitivity analysis of our primary rurality exposure. The MIZs are also used to measure rurality.^17^ MIZs distinguish those rural areas with less access to urban centre labour markets to regions with more access. If a MIZ has >10,000 people, it is considered urban.^17^ Levels of rurality are determined by those commuting to work in a census metropolitan agglomeration (CMA) or census agglomeration (CA). A MIZ is considered strong when 30% or more individuals work in CMA/CA, moderate (5–30%), weak (1–5%), and remote (≤40 people).

While both measures consider population density, the RIO captures distance to health services, while the MIZ captures labour market access; both measures are used because they capture different aspects of rurality.

### Covariates

The Johns Hopkins Adjusted Clinical Groups (ACG)® System version 10 is used to categorize patients’ chronic conditions. International disease classification diagnoses are categorized into 32 aggregate diagnosis groups (ADGs). These 32 disease classifications have been previously validated for use in predicting mortality amongst a population-based cohort of adults with schizophrenia in Ontario, Canada.^18^ These classifications are based on five clinical dimensions: duration of the condition (acute, recurrent, or chronic); severity of the condition (e.g., minor and stable versus major and unstable); diagnostic certainty (symptoms vs. documented disease); etiology of the condition (infectious or injury); and specialty care involvement (e.g., medical, surgical, obstetric).^19^ We also included migrant status, the Ontario Marginalization Indices of instability and dependency, and neighbourhood income quintile as covariates. For death by suicide, whether the individual had a prior suicide attempt is also controlled for.

### Statistical Analysis

For descriptive analyses, we compared cases and controls using standardized differences. Next, bivariate models stratified by matched groups were created to model the association between levels of rurality and the suicide outcomes. Multivariable conditional logistic regression models including all covariates and stratified by matched groups were generated for each of the two main outcomes: attempted suicide and death by suicide. Separate analyses are completed for males and females. All covariates are forced into the model to allow for comparability between effect estimates. Results are reported as odds ratios. The odds ratio approximates the risk ratio because both suicide and attempted suicides are relatively rare outcomes. Furthermore, controls were selected and matched at the index date for cases, increasing the likelihood that the control subjects represent the source population's distribution of person-time of exposure over the risk period.^20^

### Sensitivity Analysis

We did a sensitivity analysis that examined the relationship before and after the median index date to see if the relationship between rurality and suicide changes over time. We also conducted a second sensitivity analysis adjusting for deprivation index instead of neighbourhood income quintile. We also did a third sensitivity analysis where we imputed missing variables with the mean response for that variable because responses were missing for income quintile and the marginalization indices (dependency and residential instability indices) in some cases.

The analyses, conclusions, opinions, and statements expressed herein are solely those of the authors and do not reflect those of the funding or data sources.

### Ethics Committee Approval

Ethics approval for this study was obtained from the Health Sciences Research Ethics Board at The University of Toronto (Protocol number 20397).

## Results

### Descriptive Results

Of the 9,848 people who died by suicide, 2,608 (26.48%) were female (Table 1). Compared with people who did not die by suicide, people who died by suicide were more likely to be nonimmigrants, live in lower income neighbourhoods, and come from neighbourhoods with a higher instability index. People who died by suicide were more likely to have all Johns Hopkins ADG diagnoses except for time-limited primary infections; allergies; asthma; stable orthopedic; stable ear, nose, or throat and eye; unstable chronic ear, nose or throat and eye; see and reassure; prevention and administrative, where no differences were observed. Cases were less likely to be pregnant or have dermatologic diagnoses. Cases were more likely to have a prior suicide attempt.

**Table 1.** Descriptive Statistics: Suicide.

Variables	Case (*N* = 9,848), *n* (%)	Control (*N* = 39,392), *n* (%)	Standardized difference
RIO score			
Mean (SD)	12.13 (18.71)	10.15 (16.80)	0.083
RIO category			
Urban	6,734 (68.38)	28,634 (72.69)	0.095
Small urban	2,428 (24.65)	8,718 (22.13)	0.060
Rural	686 (6.97)	2,040 (5.18)	0.075
Age	48.73 (16.42)	48.74 (16.42)	−0.001
Sex (% female)	2,608 (26.48)	10,432 (26.48)	0
Immigration status			
Nonimmigrant	9,017 (91.56)	32,827 (83.33)	0.250
Immigrant	622 (6.32)	5,412 (13.74)	0.249
Refugee	209 (2.12)	1,153 (2.93)	0.051
Neighbourhood income quintile			
Q1 (lowest)	2,523 (25.83)	7,222 (18.41)	0.177
Q2	2,092 (21.42)	7,716 (19.67)	0.041
Q3	1,898 (19.43)	7,898 (20.14)	0.020
Q4	1,688 (17.28)	8,218 (20.95)	0.095
Q5 (highest)	1,565 (16.02)	8,169 (20.83)	0.126
Instability index quintile			
1 (lowest instability)	1,293 (13.37)	8,236 (21.08)	0.208
2	1,542 (15.95)	7,628 (19.52)	0.098
3	1,769 (18.30)	7,303 (18.69)	0.015
4	2,007 (20.76)	7,416 (18.98)	0.039
5 (highest instability)	3,057 (31.62)	8,489 (21.73)	0.217
Dependency quintile			
1 (lowest dependency)	2,082 (21.53)	9,389 (24.03)	0.065
2	1,833 (18.96)	7,892 (20.20)	0.036
3	1,771 (18.32)	7,436 (19.03)	0.023
4	1,865 (19.29)	6,922 (17.72)	0.035
5 (highest dependency)	2,117 (21.90)	7,433 (19.02)	0.066
Time limited minor (ADG1)	2,528 (25.67)	8,040 (20.41)	0.125
Time limited minor: primary infection (ADG2)	4,242 (43.07)	15,294 (38.83)	0.087
Time limited major (ADG3)	1,476 (14.99)	2,063 (5.24)	0.328
Time limited major: primary infection (ADG4)	1,697 (17.23)	3,346 (8.49)	0.263
Allergies (ADG5)	543 (5.51)	2,242 (5.69)	−0.007
Asthma (ADG6)	620 (6.30)	1,737 (4.41)	0.084
Likely to recur: discrete (ADG7)	3,808 (38.67)	11,361 (28.84)	0.209
Likely to recur: discrete infection (ADG8)	1,891 (19.20)	5,709 (14.49)	0.126
Likely to recur progressive (ADG9)	852 (8.65)	1,017 (2.58)	0.266
Chronic medical: stable (ADG10)	4,635 (47.07)	15,865 (40.27)	0.137
Chronic medical: unstable (ADG11)	3,194 (32.43)	7,254 (18.41)	0.326
Chronic specialty: stable-orthopedic (ADG12)	338 (3.43)	991 (2.52)	0.054
Chronic specialty: stable-ear, nose, throat (ADG13)	267 (2.71)	833 (2.11)	0.039
Chronic specialty-stable-eye (ADG14)	560 (5.69)	1,958 (4.97)	0.032
Chronic specialty: unstable-orthopedic (ADG16)	418 (4.24)	874 (2.22)	0.115
Chronic specialty: unstable-ear, nose, throat (ADG17)	<10	<10	−0.01
Chronic specialty: unstable-eye (ADG18)	579 (5.88)	2,063 (5.24)	0.028
Dermatologic (ADG20)	1,170 (11.88)	4,981 (12.64)	−0.023
Injuries/adverse effects: minor (ADG21)	3,277 (30.60)	7,431 (18.86)	0.333
Injuries/adverse effects: major (ADG22)	3,904 (39.64)	5,665 (14.38)	0.593
Psychosocial: time limited, minor (ADG23)	1,476 (14.99)	1,431 (3.63)	0.398
Psychosocial: recurrent or persistent, stable (ADG24)	6,338 (64.36)	8,440 (21.43)	0.963
Psychosocial: recurrent or persistent, unstable (ADG25)	3,755 (38.13)	2,094 (5.32)	0.867
Signs/symptoms: minor (ADG26)	4,464 (45.33)	12,071 (30.64)	0.306
Signs/symptoms: uncertain (ADG27)	5,932 (60.24)	17,291 (43.89)	0.332
Signs/symptoms: major (ADG28)	3,750 (38.08)	9,456 (24.00)	0.308
Discretionary (ADG29)	1,930 (19.60)	6,282 (15.95)	0.096
See and reassure (ADG30)	243 (2.47)	802 (2.04)	0.029
Prevention/administrative (ADG31)	3,327 (33.78)	13,150 (33.38)	0.008
Malignancy (ADG32)	928 (9.42)	2,673 (6.79)	0.097
Pregnancy (ADG33)	123 (1.25)	625 (1.59)	0.029
Dental (ADG34)	386 (3.92)	654 (1.66)	0.138
Prior suicide attempt	1,832 (18.60)	218 (0.55)	0.644

ADG: aggregate diagnosis group; RIO: Rurality Index of Ontario; SD: standard deviation.

Of the 82,180 individuals who attempted suicide, 43,024 (52.18%) were female (Table 2). Compared with people who did not attempt suicide, people who did attempt suicide were more likely to be nonimmigrants, live in lower income neighbourhoods, and come from neighbourhoods with a higher instability index. Cases were more likely to have all ADG diagnoses with the exception of allergies; stable chronic orthopedic; stable chronic ear, nose, or throat and eye; unstable chronic ear, nose, or throat and eye; prevention and administrative; malignancy; pregnancy and dermatologic diagnosis.

**Table 2.** Descriptive Statistics: Attempted Suicide.

Variables	Case (*N* = 82,480), *n* (%)	Control (*N* = 164,960), *n* (%)	Standardized difference
Rurality Index of Ontario score			
Mean (SD)	10.97 (17.77)	9.83 (16.41)	0.055
Rurality Index of Ontario category			
Urban	58,606 (71.05)	121,102 (73.41)	0.053
Small urban	19,054 (23.10)	35,883 (21.75)	0.032
Rural	4,820 (5.84)	7,975 (4.83)	0.045
Age	39.96 (16.07)	39.98 (16.06)	0.002
Sex (% female)	43,034 (52.18)	86,068 (52.18)	0
Immigration status			
Nonimmigrant	74,918 (90.83)	136,671 (82.85)	0.238
Immigrant	5,747 (6.97)	23,517 (14.26)	0.238
Refugee	1,815 (2.20)	4,772 (2.89)	0.044
Income quintile			
Q1 (lowest)	23,665 (28.94)	31,093 (18.92)	0.233
Q2	17,612 (21.54)	32,496 (19.78)	0.041
Q3	14,879 (18.20)	33,129 (20.16)	0.052
Q4	13,565 (16.59)	34,662 (21.10)	0.117
Q5 (highest)	12,044 (14.73)	32,923 (20.04)	0.142
Instability index quintile			
1	10,820 (13.42)	35,693 (21.81)	0.226
2	11,922 (14.79)	31,877 (19.48)	0.130
3	13,722 (17.02)	29,894 (18.26)	0.039
4	17,571 (21.80)	30,882 (18.87)	0.065
5	26,576 (32.97)	35,323 (21.58)	0.246
Dependency quintile			
1	18,779 (23.30)	42,475 (25.95)	0.07
2	16,203 (20.10)	34,435 (21.04)	0.031
3	15,117 (18.75)	30,743 (18.78)	0.008
4	14,691 (18.22)	28,389 (17.35)	0.016
5	15,281 (19.63)	27,627 (16.88)	0.063
Time limited minor (ADG1)	26,700 (32.37)	35,835 (21.72)	0.241
Time limited minor: primary infection (ADG2)	45,300 (54.92)	70,677 (42.84)	0.243
Time limited major (ADG3)	8,895 (10.78)	6,802 (4.12)	0.256
Time limited major : primary infection (ADG4)	14,654 (17.77)	13,033 (7.90)	0.298
Allergies (ADG5)	5,894 (7.15)	10,525 (6.38)	0.03
Asthma (ADG6)	7,241 (8.78)	7,865 (4.77)	0.16
Likely to recur: discrete (ADG7)	36,242 (43.94)	47,953 (29.07)	0.313
Likely to recur: discrete infection (ADG8)	20,847 (25.28)	28,263 (17.13)	0.200
Likely to recur progressive (ADG9)	4,538 (5.50)	2,514 (1.52)	0.217
Chronic medical: stable (ADG10)	33,834 (41.02)	50,007 (30.31)	0.225
Chronic medical: unstable (ADG11)	20,508 (24.86)	21,661 (13.13)	0.303
Chronic specialty: stable-orthopedic (ADG12)	2,822 (3.42)	3,474 (2.11)	0.080
Chronic specialty: stable-ear, nose, throat (ADG13)	1,605 (1.95)	2,551 (1.55)	0.031
Chronic specialty-stable-eye (ADG14)	3,415 (4.14)	5,107 (3.10)	0.056
Chronic specialty: unstable-orthopedic (ADG16)	3,355 (4.07)	2,666 (1.62)	0.148
Chronic specialty: unstable-ear, nose, throat (ADG17)	10 (0.01)	<10	0.010
Chronic specialty: unstable-eye (ADG18)	3,547 (4.30)	6,330 (3.84)	0.023
Dermatologic (ADG20)	10,719 (13.00)	22,088 (13.39)	−0.011
Injuries/adverse effects: minor (ADG21)	31,290 (37.94)	31,239 (18.94)	0.431
Injuries/adverse effects: major (ADG22)	57,928 (70.23)	22,422 (13.59)	1.402
Psychosocial: time limited, minor (ADG23)	15,605 (18.92)	5,973 (3.62)	0.499
Psychosocial: recurrent or persistent, stable (ADG24)	62,399 (75.65)	37,009 (22.44)	1.258
Psychosocial: recurrent or persistent, unstable (ADG25)	36,436 (44.18)	6,701 (4.06)	1.060
Signs/symptoms: minor (ADG26)	40,856 (49.53)	49,810 (30.20)	0.403
Signs/symptoms: uncertain (ADG27)	54,244 (65.77)	71,015 (43.05)	0.468
Signs/symptoms: major (ADG28)	34,397 (41.70)	42,303 (25.64)	0.345
Discretionary (ADG29)	16,764 (20.32)	23,837 (14.45)	0.155
See and reassure (ADG30)	1,692 (2.05)	2,495 (1.51)	0.041
Prevention/administrative (ADG31)	31,231 (37.86)	61,184 (37.09)	0.016
Malignancy (ADG32)	4,794 (5.81)	7,190 (4.36)	0.066
Pregnancy (ADG33)	3,377 (4.09)	7,598 (4.61)	−0.025
Dental (ADG34)	4,579 (5.55)	2,778 (1.68)	0.208

ADG: aggregate diagnosis group; SD: standard deviation.

### Death by Suicide

**Figure 1.**
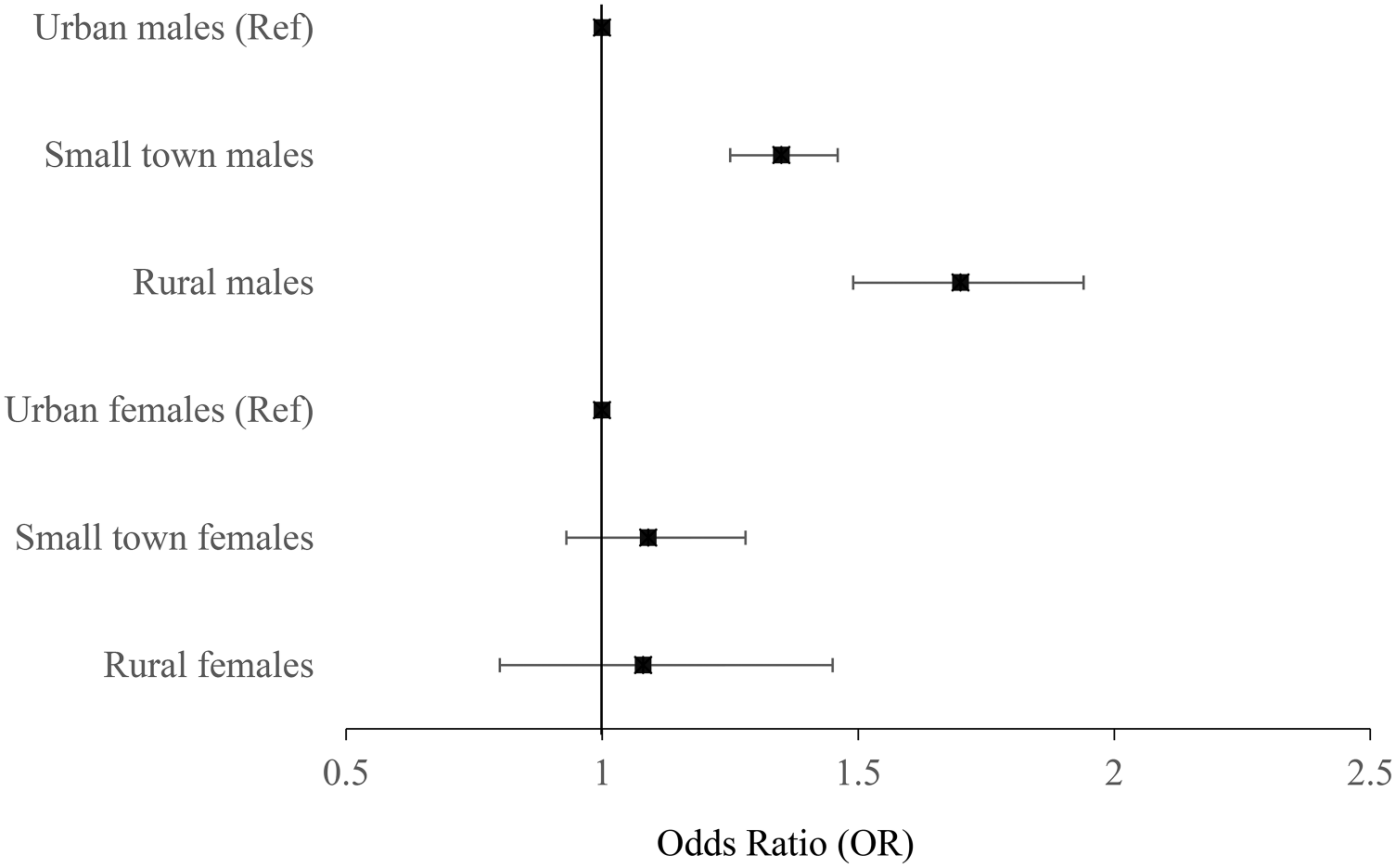
Fully adjusted effect estimates for death by suicide.

Both the unadjusted and fully adjusted models (Table 3, Figure 1) indicate that rural males were significantly more likely to die by suicide than urban males (AOR = 1.70, 95% confidence interval (CI), 1.49 to 1.94). Males in small towns and in rural areas were more likely to die by suicide than urban males. Using the MIZ as an exposure (Table S3) also indicates that rural males were at increased risk of suicide compared with urban males (AOR = 2.14, 95% CI, 1.74 to 2.63). Rural females were not significantly more likely to die by suicide than their urban counterparts using the MIZ or the RIO.

**Table 3.** Adjusted and Unadjusted Associations Between Rurality and Suicide Outcomes. Odds Ratios (OR).

Exposure	Death by suicide: male	Death by suicide: female	Suicide attempt: male	Suicide attempt: female
Unadjusted model				
Urban (reference)	Reference	Reference	Reference	Reference
Small town	1.26 (1.18, 1.34)	1.00 (0.90, 1.11)	1.10 (1.07, 1.13)	1.10 (1.07, 1.13)
Rural	1.62 (1.46, 1.79)	0.98 (0.81, 1.20)	1.24 (1.17, 1.31)	1.26 (1.20, 1.33)
Fully adjusted model^a^				
Urban (reference)	Reference	Reference	Reference	Reference
Small town	1.35 (1.25, 1.46)	1.09 (0.93, 1.28)	1.15 (1.09, 1.21)	1.13 (1.07, 1.19)
Rural	1.70 (1.49, 1.94)	1.08 (0.80, 1.45)	1.37 (1.24, 1.50)	1.26 (1.14, 1.39)

*Note:* ^a^Adjusted for income quintile, immigration status, dependency and instability marginalization indices and Johns Hopkins aggregate diagnosis groups. Adjusted for prior suicide attempt for death by suicide.

### Attempted Suicide

**Figure 2.**
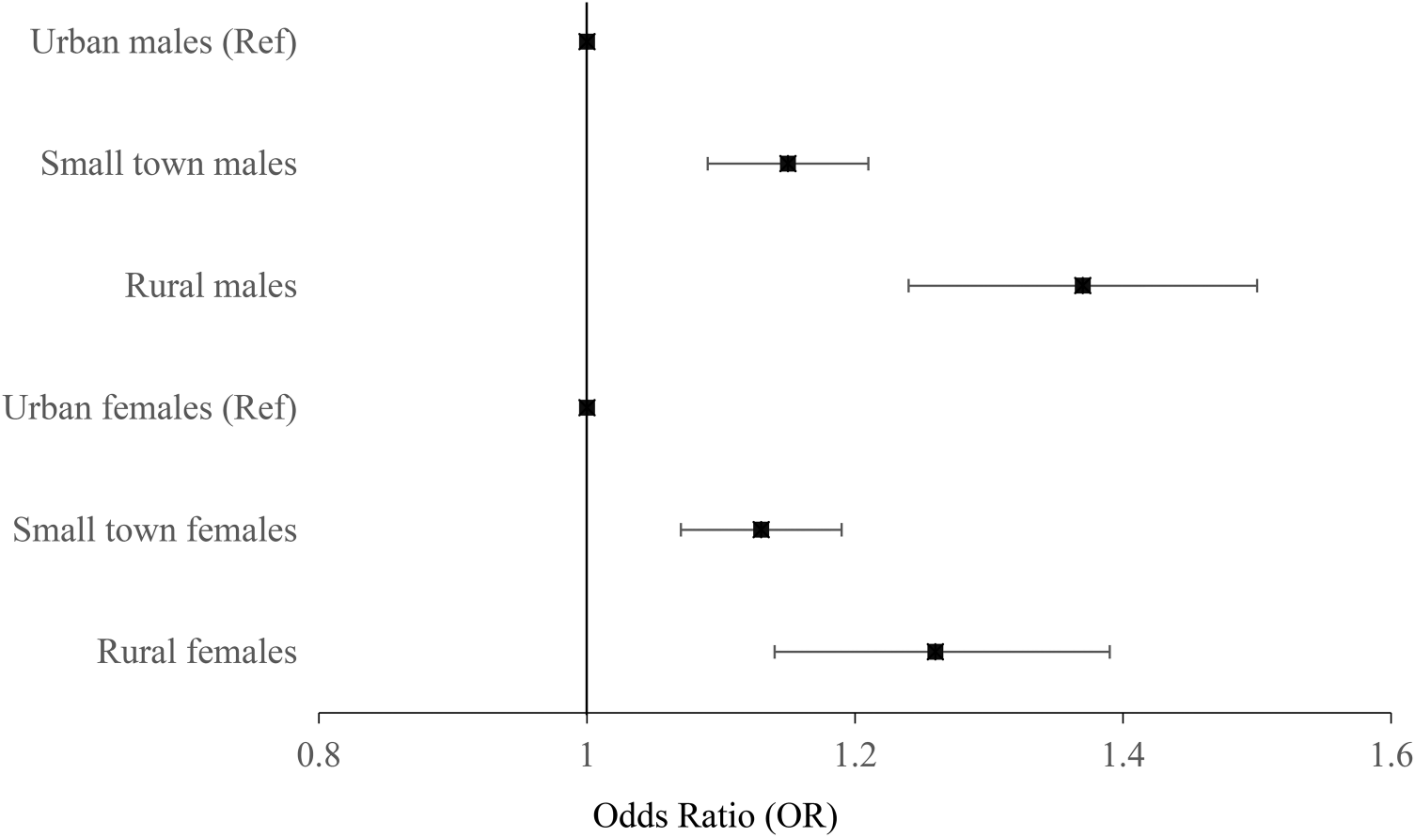
Fully adjusted effect estimates for attempted suicide.

Rural males were significantly more likely to attempt suicide than their urban counterparts; this is significant for both the RIO and MIZ as measures of rurality. Table 3 and Figure 2 show that when considering the RIO, males from rural areas were 1.37 (95% CI, 1.24 to 1.50) times more likely to attempt suicide, while those living in small towns were 1.15 (95% CI, 1.09 to 1.21) times more likely to attempt suicide. Males in the most rural MIZ category were 1.73 (95% CI, 1.49 to 2.00) times more likely to attempt suicide than the most urban males (Table S3).

Rural females were also significantly more likely to attempt suicide than their urban counterparts. Using the RIO, rural and small town females were 1.26 (95% CI, 1.14 to 1.39) and 1.13 (95% CI, 1.07 to 1.19) times more likely to attempt suicide, respectively. Using the MIZ, the most rural females were 1.45 (95% CI, 1.34 to 1.68) times more likely to attempt suicide than the most urban females based on MIZ as the exposure. Females at all levels of increasing rurality were more likely to die by suicide than the urban females, with most effect estimates increasing as rurality increases.

### Sensitivity Analysis

Findings appear relatively consistent over time when comparing those who died by suicide or attempted suicide prior to the median index date versus those after the median date (Table S5). The point estimates comparing those living in the most rural areas with those living in the most urban are generally higher for the more recent dates within the study period, indicating that, if anything, this disparity is increasing over time. Analyses controlling for neighbourhood deprivation indices instead of neighbourhood income quartile did not change results. Analyses that imputed missing responses for income quintile and the marginalization indices, results are similar when using the RIO as the exposure (Table S6). When using the MIZ as an exposure, the results indicate that those in the most rural areas may be at even greater risk of suicide and also indicate that the most rural females may be more likely to die by suicide (AOR = 2.19. 95% CI, 1.52 to 3.14) (Table S4.6).

Overall, rurality is associated with an increased odds of death by suicide and suicide attempts amongst males and increased odds of suicide attempts amongst females. Therefore, there is a significant increase in risk of suicide, with the most rural males being 70% more likely to die by suicide than urban males, and 37% more likely to attempt suicide. Females living in rural areas have a 26% increase in risk of attempting suicide compared with females living in the most urban areas. When using the MIZ, the most rural males are over two times more likely to die by suicide than the most urban males, while the most rural males and most rural females are 75% and 45% more likely to attempt suicide, respectively. The effect estimates also indicate a relationship where suicide and suicide attempts become more likely with increasing levels of rurality.

## Discussion

One reason why suicides may be higher in rural areas is access to healthcare. There may be less specialized psychiatric care available in rural areas compared with larger urban centres. People living in rural areas may need to travel further to access primary and psychiatric care, and lengthy travel times or lack of transportation may result in lower rates of seeking help. Stigma may also contribute to differences in help-seeking behaviours.^21^

It is also possible that differences in suicide methods may explain this rural–urban gradient in suicidal behaviour. For example, previous research has shown that those living in rural areas are more likely to use firearms as a means of suicide compared with people living in urban areas who are more likely to use poisoning.^5,22^ Firearm suicides may be more likely to be reported as a suicide compared with poisoning, because poisoning may be interpreted as an accidental overdose. However, this would only explain differences for death by suicide and not suicide attempts.

There may also be other social determinants of health that affect those living in rural areas and may contribute to suicidal behaviour. There is lower employment growth, lower percentages of workers with postsecondary credentials, and higher poverty rates in rural areas.^23-25^ Occupational hazards differ between rural and urban areas, and general mortality due to injury is higher.^26^ While illicit drug use appears to be more common in urban areas, drug overdose death rates have been increasing in rural areas over time and have recently surpassed metropolitan areas.^27^ Students who attend rural schools are more likely to drink alcohol, binge drink, and drive under the influence of drugs or alcohol compared to their urban counterparts.^28^ Infrastructures such as roads, transportation options, telecommunication availability, and availability of health services differ between rural and urban areas.^29^ These social determinants may be driving rural-urban differences in suicidality.

One possible explanation for the sex effect modification is that cultural norms and attitudes towards masculinity in a rural setting may inhibit help-seeking amongst rural males for mental health issues.^30^ Another possible explanation is that males in rural areas are likely to be employed in occupations such as farming or forestry, which are associated with higher risk of suicide.^31^ A third explanation is that there is less access to care in rural areas, and this lack of access may be more detrimental to males in rural areas because emotionally supportive relationships are substantially more protective against major depression for women than for men.^32^

This study has several strengths. First, this population-based case-control study uses a data source that captures health care service use for almost all people living in the province of Ontario, which decreases potential for selection bias. No studies from the United States published since 2006 use a population-based cohort to examine this association, and while one study from Canada does, it uses data from 1991 to 2001.^5^ Second, the data capture the exposure information prior to the outcome. As the outcome cannot retroactively affect data collection of exposure information, this eliminates the risk of the outcome measurement affecting the exposure measurement. This establishes temporality, which is an important indicator of causality. Third, all data are collected by medical staff rather than self-reported. This minimizes the potential for response bias including social desirability bias and recall bias. Fourth, data quality is considered to be high because most of the data are routinely and systematically collected and undergoes quality assessment by data quality analysts. Fifth, Ontario consists of very rural and remote areas (particularly in Northern Ontario) in addition to large urban centers such as Toronto. This provides a large degree of variability within the main exposure of interest. Finally, the sample size is large, and although suicide is a rare outcome in the general population, we have the power to detect smaller differences between groups. This study also has several limitations. First, some potential mediators of the association between rurality and suicide cannot be studied because the administrative data do not have information on these factors. These potential mediators include stigma, health beliefs, and attitudes towards help-seeking. A second limitation is that we do not have access to Indigenous status, which may be an effect modifier of the association between rural residence and suicide attempt and suicide completion. A recent report indicates that suicide rates amongst First Nations in Canada are about three times higher than amongst non-Indigenous people, while suicide rates among Inuit are nine times higher and suicide rates among Métis are twice as high.^29^ Furthermore, First Nations, Métis, and Inuit people disproportionately live in rural areas.^29^ Therefore, it is possible that Indigenous status is a potential effect modifier of the association between rurality and suicide. Another potential effect modifier is lesbian, gay, bisexual, transgender, and queer (LGBTQ) status. Sexual minority adults have higher risk of suicide attempts and death by suicide than heterosexual adults.^33,34^ Furthermore, sexual minority adults living in rural areas report higher levels of depression and suicidal behaviour than sexual minority adults living in more urban areas and may experience more barriers to mental healthcare.^35-37^ A third limitation is that suicide mortality may be underestimated as some suicides may be recorded as accidental, and this misclassification may differ by urban/rural residence. Those in rural areas are more likely to use firearms to end their life, while those in more urban areas are more likely to use drugs, which may be more likely to be considered an accidental death.^38^ Drugs are also less fatal than firearms, but this can be accounted for by examining suicide attempts in addition to deaths by suicide.^38^ A fourth limitation is that the MIZ scores and RIO scores are based on census data collected in 2011. Some individuals may have moved from rural to urban areas or vice versa, which introduces noise and thus bias towards the null. Finally, there may be additional unmeasured confounding factors that increase risk of suicide and may also increase the likelihood of someone choosing to reside in a rural area that may not be captured in our data.

## Conclusion

Our findings indicate a major increase in suicidal risk and behaviour in rural regions. Future research needs to determine which factors mediate the association between rurality and suicide, paying particular attention to help-seeking behaviours and access to care prior to suicide or suicide attempt. These factors can then be used to inform policy or initiate interventions. Potential interventions include increased access to telephone-based mental health care services, internet-based mental health care services (and policy changes to ensure high-speed internet for adequate streaming), community-based approaches that involve working with existing health care resources, and targeted mental health and suicide training for general practitioners practising in high risk areas. Given the higher likelihood to die by suicide amongst males, more needs to be done to address the high rate of suicide and suicide attempts among rural males.

## Data Access

The data set from this study is held securely in coded form at ICES. While data sharing agreements prohibit ICES from making the data set publicly available, access may be granted to those who meet prespecified criteria for confidential access, available at www.ices.on.ca/DAS.

## Supplemental Material

sj-docx-1-cpa-10.1177_07067437211053300 - Supplemental material for Rurality as a Risk Factor for Attempted Suicide and Death by Suicide in Ontario, Canada Click here for additional data file. Supplemental material, sj-docx-1-cpa-10.1177_07067437211053300 for Rurality as a Risk Factor for Attempted Suicide and Death by Suicide in Ontario, Canada by Rebecca Barry, Jürgen Rehm, Claire de Oliveira, Peter Gozdyra, Simon Chen and Paul Kurdyak in The Canadian Journal of Psychiatry
