# Supplementary material for: Rurality as a Risk Factor for Attempted Suicide and Death by Suicide in Ontario, Canada
Source: Can J Psychiatry. 2021 Nov 18;67(9):679–89. doi: 10.1177/07067437211053300 (PMC9449140; doi:10.1177/07067437211053300)
Supplement: sj-docx-1-cpa-10.1177_07067437211053300 - Supplemental material for Rurality as a Risk Factor for Attempted Suicide and Death by Suicide in Ontario, Canada [file sj-docx-1-cpa-10.1177_07067437211053300.docx]

eTable 1: Descriptive Statistics: Metropolitan Influence Zones

| Suicide | Case (N=9848) | Control (N=39392) | Standardized Difference |
| --- | --- | --- | --- |
| Metropolitan Influence Zone |  |  |  |
| Urban: POP 1, 500, 000+ | 3389 (34.41) | 16758 (42.54) | 0.168 |
| Urban: POP 500, 000-1,499, 999 | 1211 (12.30) | 4886 (12.40) | 0.003 |
| Urban: POP 100, 000-499, 999 | 2706 (27.48) | 9850 (25.01) | 0.056 |
| Urban: POP 10, 000-99, 999 | 1016 (10.32) | 3224 (8.18) | 0.074 |
| Rural: Strong MIZ | 612 (6.21) | 1972 (5.01) | 0.053 |
| Rural: Moderate MIZ | 566 (5.75) | 1856 (4.71) | 0.047 |
| Rural: Weak MIZ | 348 (3.53) | 846 (2.15) | 0.084 |
| Suicide | Case (N=82480) | Control (N=164960) | Standardized Difference |
| Metropolitan Influence Zone |  |  |  |
| Urban: POP 1, 500, 000+ | 28522 (34.57) | 70837 (42.94) | 0.172 |
| Urban: POP 500, 000-1,499, 999 | 9581 (11.62) | 20827 (12.63) | 0.031 |
| Urban: POP 100, 000-499, 999 | 24616 (29.84) | 41597 (25.22) | 0.104 |
| Urban: POP 10, 000-99, 999 | 9154 (11.10) | 13187 (7.99) | 0.106 |
| Rural: Strong MIZ | 3686 (4.47) | 8293 (5.03) | 0.026 |
| Rural: Moderate MIZ | 3789 (4.59) | 6744 (4.09) | 0.025 |
| Rural: Weak MIZ | 3132 (3.80) | 3475 (2.11) | 0.100 |

eTable 2: Unadjusted bivariate associations between rurality and suicide outcomes

| Exposure | Suicide: Male | Suicide: Female | Suicide Attempt: Male | Suicide Attempt: Female |
| --- | --- | --- | --- | --- |
| **Metropolitan Influence Zones** |  |  |  |  |
| Urban: POP 1, 500, 000+ | Ref | Ref | Ref | Ref |
| Urban: POP 500, 000-1,499, 999 | 1.24 (1.14, 1.36) | 1.19 (1.03, 1.36) | 1.08 (1.04, 1.13) | 1.20 (1.15, 1.25) |
| Urban: POP 100, 000-499, 999 | 1.42 (1.33, 1.52) | 1.20 (1.08, 1.34) | 1.40 (1.36, 1.45) | 1.53 (1.49, 1.58) |
| Urban: POP 10, 000-99, 999 | 1.65 (1.51, 1.82) | 1.35 (1.15, 1.58) | 1.74 (1.67, 1.82) | 1.71 (1.64, 1.79) |
| Rural: Strong MIZ | 1.70 (1.52, 1.90) | 1.16 (0.94, 1.42) | 1.06 (1.00, 1.13) | 1.15 (1.09, 1.22) |
| Rural: Moderate MIZ | 1.73 (1.54, 1.94) | 0.98 (0.79, 1.22) | 1.33 (1.25, 1.41) | 1.46 (1.38, 1.55) |
| Rural: Weak MIZ | 2.15 (1.84, 2.50) | 1.82 (1.41, 2.35) | 2.15 (2.00, 2.31) | 2.34 (2.18, 2.51) |

eTable 3: Fully adjusted models using Metropolitan Influence Zones as Exposure

| Variable | Suicide: Male | Suicide: Female | Suicide Attempt: Male | Suicide Attempt: Female |
| --- | --- | --- | --- | --- |
| Metropolitan Influence Zones |  |  |  |  |
| Urban: POP 1, 500, 000+ | Ref | ref | Ref | Ref |
| Urban: POP 500, 000-1,499, 999 | 1.07 (0.96, 1.19) | 1.15 (0.94, 1.41) | 0.78 (0.73, 0.84) | 0.91 (0.85. 0.98) |
| Urban: POP 100, 000-499, 999 | 1.20 (1.10, 1.30) | 0.94 (0.79, 1.11) | 0.92 (0.87, 0.97) | 1.04 (0.99, 1.10) |
| Urban: POP 10, 000-99, 999 | 1.45 (1.29, 1.64) | 1.09 (0.85, 1.40) | 1.26 (1.16, 1.36) | 1.26 (1.16, 1.36) |
| Rural: Strong MIZ | 1.76 (1.53, 2.03) | 1.38 (1.00, 1.90) | 1.03 (0.93, 1.14) | 1.19 (1.07, 1.33) |
| Rural: Moderate MIZ | 1.83 (1.58, 2.12) | 1.37 (1.00, 1.90) | 1.10 (0.99, 1.22) | 1.30 (1.16, 1.45) |
| Rural: Weak MIZ | 2.14 (1.74, 2.63) | 1.02 (0.64, 1.63) | 1.73 (1.49, 2.00) | 1.45 (1.34, 1.68) |
| Income quintile |  |  |  |  |
| 1 | 1.33 (1.19, 1.49) | 1.17 (0.95, 1.45) | 1.43 (1.33, 1.53) | 1.31 (1.21, 1.41) |
| 2 | 1.25 (1.13, 1.39) | 1.00 (0.82, 1.23) | 1.20 (1.12, 1.29) | 1.21 (1.13, 1.30) |
| 3 | 1.23 (1.11, 1.37) | 0.99 (0.81, 1.32) | 1.16 (1.08, 1.24) | 1.10 (1.03, 1.18) |
| 4 | 1.07 (0.96, 1.18) | 1.01 (0.82, 1.25) | 1.09 (1.01, 1.16) | 1.05 (0.98, 1.13) |
| 5 | Ref | Ref | Ref | Ref |
| Immigrant status |  |  |  |  |
| Non-immigrant | Ref | Ref | Ref | Ref |
| Immigrant | 0.55 (0.48, 0.62) | 0.78 (0.63, 0.98) | 0.68 (0.63, 0.73) | 0.88 (0.82, 0.95) |
| Refugee | 0.75 (0.61, 0.92) | 0.71 (0.45, 1.11) | 0.76 (0.67, 0.86) | 1.05 (0.91, 1.20) |
| Dependency | 0.99 (0.96, 1.03) | 0.94 (0.88, 1.01) | 0.98 (0.95, 1.00) | 0.96 (0.94, 0.98) |
| Instability | 1.11 (1.07, 1.15) | 1.25 (1.18, 1.34) | 1.18 (1.15, 1.20) | 1.16 (1.14, 1.19) |
| Time Limited Minor (ADG1) | 0.94 (0.86, 1.02) | 0.85 (0.73, 0.98) | 1.10 (1.04, 1.16) | 1.14 (1.08, 1.19) |
| Time Limited Minor: Primary Infection (ADG2) | 0.81 (0.75, 0.87) | 0.74 (0.65, 0.85) | 0.97 (0.92, 1.01) | 0.98 (0.93, 1.02) |
| Time Limited Major (ADG3) | 1.34 (1.18, 1.52) | 1.41 (1.12, 1.78) | 1.15 (1.04, 1.27) | 1.16 (1.06, 1.27) |
| Time Limited Major : Primary Infection (ADG4) | 1.09 (0.98, 1.21) | 1.13 (0.93, 1.38) | 1.16 (1.08, 1.24) | 1.11 (1.04, 1.19) |
| Allergies (ADG5) | 0.86 (0.74, 1.01) | 0.88 (0.68, 1.12) | 0.83 (0.75, 0.91) | 0.91 (0.84, 0.98) |
| Asthma (ADG6) | 0.98 (0.84, 1.15) | 1.07 (0.84, 1.38) | 1.02 (0.93, 1.13) | 1.06 (0.98, 1.15) |
| Likely to recur: discrete (ADG7) | 0.93 (0.86, 1.00) | 1.07 (0.93, 1.24) | 1.02 (0.93, 1.13) | 1.04 (0.99, 1.09) |
| Likely to recur: discrete infection (ADG8) | 0.99 (0.90, 1.10) | 0.88 (0.75, 1.03) | 0.90 (0.84, 0.96) | 1.07 (1.02, 1.13) |
| Likely to recur Progressive (ADG9) | 1.28 (1.10, 1.50) | 1.61 (1.17, 2.20) | 1.30 (1.14, 1.48) | 1.21 (1.06, 1.39) |
| Chronic Medical: Stable (ADG10) | 0.86 (0.80, 0.93) | 0.90 (0.77, 1.04) | 0.91 (0.86, 0.97) | 0.90 (0.85, 0.95) |
| Chronic Medical: Unstable (ADG11) | 1.16 (1.06, 1.27) | 1.31 (1.12, 1.54) | 1.06 (1.00, 1.14) | 1.07 (1.01, 1.14) |
| Chronic Specialty: Stable-Orthopedic (ADG12) | 0.78 (0.64, 0.94) | 0.74 (0.50, 1.08) | 0.49 (0.43, 0.55) | 0.60 (0.53, 0.69) |
| Chronic Specialty: Stable-Ear, Nose, Throat (ADG13) | 1.05 (0.85, 1.28) | 0.91 (0.59, 1.41) | 0.81 (0.69, 0.96) | 0.86 (0.73, 1.00) |
| Chronic Specialty-Stable-Eye (ADG14) | 0.93 (0.80, 1.09) | 0.90 (0.68, 1.19) | 1.11 (0.98, 1.27) | 0.96 (0.86, 1.08) |
| Chronic Specialty: Unstable-Orthopedic (ADG16) | 1.03 (0.85, 1.24) | 1.17 (0.84, 1.64) | 1.11 (0.96, 1.27) | 1.12 (0.98, 1.28) |
| Chronic Specialty: Unstable-Ear, Nose, Throat (ADG17) | 0.97 (0.03, 31.66) | 0.97 (0.03, 31.66) | 0.99 (0.03, 36.34) | 0.99 (0.18, 5.47) |
| Chronic Specialty: Unstable-Eye (ADG18) | 1.02 (0.88, 1.18) | 1.11 (0.85, 1.46) | 0.93 (0.82, 1.04) | 0.96 (0.86, 1.08) |
| Dermatologic (ADG20) | 0.77 (0.69, 0.85) | 0.88 (0.73, 1.05) | 0.73 (0.68, 0.78) | 0.76 (0.72, 0.81) |
| Injuries/Adverse effects: Minor (ADG21) | 1.25 (1.16, 1.26) | 1.36 (1.17, 1.58) | 1.39 (1.32, 1.46) | 1.19 (1.13, 1.25) |
| Injuries/Adverse effects: Major (ADG22) | 1.50 (1.38, 1.63) | 1.99 (1.70, 2.33) | 8.38 (7.98, 8.80) | 10.87 (10.33, 11.44) |
| Psychosocial: Time Limited, Minor (ADG23) | 1.65 (1.44, 1.88) | 1.67 (1.34, 2.08) | 2.33 (2.14, 2.54) | 2.35 (2.19, 2.53) |
| Psychosocial: Recurrent or Persistent, Stable (ADG24) | 3.57 (3.32, 3.83) | 4.07 (3.53, 4.69) | 4.67 (3.35, 4.90) | 6.30 (6.00, 6.61) |
| Psychosocial: Recurrent or Persistent, Unstable (ADG25) | 3.62 (3.30, 3.97) | 5.37 (4.50, 6.41) | 6.40 (6.00, 6.82) | 7.46 (6.98, 7.97) |
| Signs/Symptoms: Minor (ADG26) | 1.08 (1.00, 1.17) | 1.19 (1.03, 1.37) | 1.26 (1.20, 1.32) | 1.24 (1.19, 1.30) |
| Signs/Symptoms: Uncertain (ADG27) | 1.12 (1.04, 1.21) | 1.12 (0.97, 1.30) | 1.34 (1.27, 1.40) | 1.20 (1.14, 1.26) |
| Signs/Symptoms: Major (ADG28) | 1.08 (1.00, 1.18) | 1.17 (1.01, 1.35) | 1.15 (1.08, 1.21) | 1.03 (0.98, 1.08) |
| Discretionary (ADG29) | 0.94 (0.85, 1.02) | 1.00 (0.85, 1.17) | 0.91 (0.85, 0.96) | 0.90 (0.85, 0.95) |
| See and Reassure (ADG30) | 1.09 (0.88, 1.35) | 0.94 (0.63, 1.39) | 0.81 (0.68, 0.97) | 0.79 (0.68, 0.92) |
| Prevention/Administrative (ADG31) | 0.78 (0.72, 0.84) | 0.77 (0.67, 0.89) | 0.78 (0.74, 0.83) | 0.78 (0.74, 0.81) |
| Malignancy (ADG32) | 1.32 (1.17, 1.50) | 0.94 (0.74, 1.19) | 0.96 (0.86, 1.07) | 0.88 (0.80, 0.96) |
| Pregnancy (ADG33) | 1.01 (0.40, 2.60) | 0.74 (0.54, 1.03) | 1.07 (0.59, 1.96) | 0.64 (0.59, 0.70) |
| Dental (ADG34) | 1.11 (0.90, 1.35) | 1.25 (0.86, 1.84) | 1.34 (1.18, 1.52) | 1.38 (1.21, 1.56) |

eTable 4: Fully adjusted models using Rurality Index of Ontario as Exposure

| Variable | Suicide: Male | Suicide: Female | Suicide Attempt: Male | Suicide Attempt: Female |
| --- | --- | --- | --- | --- |
| Rurality Index of Ontario |  |  |  |  |
| Urban (ref) | Ref | Ref | Ref | Ref |
| Small town | 1.35 (1.25, 1.46) | 1.09 (0.93, 1.28) | 1.15 (1.09, 1.21) | 1.13 (1.07, 1.19) |
| Rural | 1.70 (1.49, 1.94) | 1.08 (0.80, 1.45) | 1.37 (1.24, 1.50) | 1.26 (1.14, 1.39) |
| Income quintile |  |  |  |  |
| 1 | 1.37 (1.23, 1.53) | 1.19 (0.96, 1.47) | 1.42 (1.32, 1.52) | 1.32 (1.23, 1.42) |
| 2 | 1.27 (1.15, 1.41) | 1.01 (0.82, 1.25) | 1.19 (1.11, 1.28) | 1.22 (1.14, 1.31) |
| 3 | 1.25 (1.12, 1.38) | 1.00 (0.81, 1.23) | 1.15 (1.07, 1.24) | 1.11 (1.03, 1.19) |
| 4 | 1.06 (0.95, 1.18) | 1.02 (0.82, 1.25) | 1.08 (1.01, 1.16) | 1.04 (0.98, 1.13) |
| 5 | Ref | Ref | Ref | Ref |
| Immigrant status |  |  |  |  |
| Non-immigrant | Ref | Ref | Ref | Ref |
| Immigrant | 0.52 (0.46, 0.60) | 0.77 (0.62, 0.96) | 0.71 (0.65, 0.76) | 0.87 (0.81, 0.94) |
| Refugee | 0.72 (0.59, 0.89) | 0.71 (0.46, 1.12) | 0.78 (0.68, 0.88) | 0.97 (0.95, 0.99) |
| Dependency | 1.00 (0.907, 1.04) | 0.95 (0.89, 1.01) | 0.98 (0.96, 1.00) | 0.97 (0.95, 0.99) |
| Instability | 1.11 (1.07, 1.15) | 1.24 (1.17, 1.33) | 1.18 (1.15, 1.20) | 1.16 (1.13, 1.19) |
| Time Limited Minor (ADG1) | 0.93 (0.86, 1.01) | 0.85 (0.73, 0.98) | 1.10 (1.05, 1.16) | 1.14 (1.08, 1.19) |
| Time Limited Minor: Primary Infection (ADG2) | 0.80 (0.75, 0.86) | 0.74 (0.65, 0.85) | 0.96 (0.92, 1.01) | 0.98 (0.93, 1.02) |
| Time Limited Major (ADG3) | 1.33 (1.17, 1.51) | 1.42 (1.13, 1.80) | 1.15 (1.04, 1.27) | 1.17 (1.07, 1.27) |
| Time Limited Major : Primary Infection (ADG4) | 1.09 (0.98, 1.21) | 1.14 (0.93, 1.39) | 1.16 (1.08, 1.24) | 1.11 (1.04, 1.19) |
| Allergies (ADG5) | 0.85 (0.75, 1.00) | 0.88 (0.68, 1.12) | 0.83 (0.75, 0.91) | 0.91 (0.84, 0.98) |
| Asthma (ADG6) | 0.98 (0.84, 1.15) | 1.08 (0.85, 1.39) | 1.03 (0.93, 1.13) | 1.06 (0.97, 1.14) |
| Likely to recur: discrete (ADG7) | 0.93 (0.86, 1.00) | 1.07 (0.93, 1.23) | 1.02 (0.97, 1.08) | 1.04 (0.99, 1.09) |
| Likely to recur: discrete infection (ADG8) | 1.00 (0.90, 1.10) | 0.88 (0.75, 1.02) | 1.02 (0.97, 1.08) | 1.08 (1.03, 1.13) |
| Likely to recur Progressive (ADG9) | 1.33 (1.17, 1.51) | 1.62 (1.19, 2.22) | 1.30 (1.15, 1.48) | 1.22 (1.06, 1.39) |
| Chronic Medical: Stable (ADG10) | 0.85 (0.80, 0.93) | 0.89 (0.77, 1.04) | 0.92 (0.87, 0.97) | 0.90 (0.85, 0.95) |
| Chronic Medical: Unstable (ADG11) | 1.16 (1.06, 1.27) | 1.31 (1.12, 1.54) | 1.07 (1.00, 1.14) | 1.08 (1.01, 1.14) |
| Chronic Specialty: Stable-Orthopedic (ADG12) | 0.78 (0.64, 0.94) | 0.74 (0.51, 1.09) | 0.49 (0.43, 0.55) | 0.60 (0.53, 0.69) |
| Chronic Specialty: Stable-Ear, Nose, Throat (ADG13) | 1.04 (0.85, 1.28) | 0.92 (0.59, 1.41) | 0.81 (0.68, 0.95) | 0.86 (0.73, 1.00) |
| Chronic Specialty-Stable-Eye (ADG14) | 0.93 (0.90, 1.09) | 0.91 (0.69, 1.20) | 1.10 (0.97, 1.26) | 0.96 (0.85, 1.07) |
| Chronic Specialty: Unstable-Orthopedic (ADG16) | 1.03 (0.85, 1.24) | 1.18 (0.85, 1.66) | 1.11 (0.97, 1.28) | 1.12 (0.98, 1.28) |
| Chronic Specialty: Unstable-Ear, Nose, Throat (ADG17) | 0.98 (0.03, 30.29) | NA | 0.99 (0.03, 34.27) | 0.99 (0.18, 5.61) |
| Chronic Specialty: Unstable-Eye (ADG18) | 1.02 (0.88, 1.18) | 1.12 (0.86, 1.46) | 0.93 (0.82, 1.04) | 0.96 (0.86, 1.06) |
| Dermatologic (ADG20) | 0.76 (0.68, 0.84) | 0.87 (0.72, 1.04) | 0.73 (0.68, 0.78) | 0.76 (0.71, 0.80) |
| Injuries/Adverse effects: Minor (ADG21) | 1.26 (1.16, 1.36) | 1.36 (1.17, 1.57) | 1.39 (1.32, 1.46) | 1.19 (1.13, 1.25) |
| Injuries/Adverse effects: Major (ADG22) | 1.51(1.39, 1.64) | 1.98 (1.69, 2.32) | 8.31 (7.91, 8.72) | 10.87 (10.33, 11.43) |
| Psychosocial: Time Limited, Minor (ADG23) | 1.66 (1.46, 1.89) | 1.66 (1.33, 2.06) | 2.32 (2.13, 2.53) | 2.35 (2.19, 2.53) |
| Psychosocial: Recurrent or Persistent, Stable (ADG24) | 3.55 (3.31, 3.82) | 4.09 (3.55, 4.71) | 4.65 (4.43, 4.88) | 6.29 (6.00, 6.60) |
| Psychosocial: Recurrent or Persistent, Unstable (ADG25) | 3.59 (3.27, 3.94) | 5.32 (4.46, 6.35) | 6.39 (6.00, 6.81) | 7.45 (6.98, 7.96) |
| Signs/Symptoms: Minor (ADG26) | 1.08 (1.00, 1.16) | 1.18 (1.03, 1.36) | 1.26 (1.20, 1.32) | 1.24 (1.18, 1.30) |
| Signs/Symptoms: Uncertain (ADG27) | 1.12 (1.04, 1.21) | 1.12 (0.96, 1.30) | 1.34 (1.28, 1.41) | 1.20 (1.15, 1.26) |
| Signs/Symptoms: Major (ADG28) | 1.08 (0.99, 1.17) | 1.17 (1.01, 1.35) | 1.15 (1.08, 1.22) | 1.03 (0.98, 1.08) |
| Discretionary (ADG29) | 0.94 (0.86, 1.03) | 1.00 (0.85, 1.17) | 0.91 (0.85, 0.96) | 0.90 (0.85, 0.95) |
| See and Reassure (ADG30) | 1.10 (0.89, 1.37) | 0.94 (0.63, 1.30) | 0.81 (0.67, 0.97) | 0.80 (0.69, 0.93) |
| Prevention/Administrative (ADG31) | 0.77 (0.72, 0.84) | 0.77 (0.67, 0.88) | 0.79 (0.75, 0.83) | 0.77 (0.74, 0.81) |
| Malignancy (ADG32) | 1.33 (1.17, 1.50) | 0.94 (0.74, 1.19) | 0.95 (0.85, 1.06) | 0.87 (0.79, 0.96) |
| Pregnancy (ADG33) | 1.01 (0.40, 2.57) | 0.74 (0.54, 1.03) | 1.08 (0.59, 1.96) | 0.65 (0.60, 0.70) |
| Dental (ADG34) | 1.11 (0.91, 1.36) | 1.24 (0.85, 1.82) | 1.36 (1.20, 1.54) | 1.39 (1.23, 1.58) |

eTable 5: Sensitivity Analysis: Comparison of Adjusted Point Estimates Over Time

| Exposure | Suicide: Male | | Suicide: Female | | Suicide Attempt: Male | | Suicide Attempt: Female | |
| --- | --- | --- | --- | --- | --- | --- | --- | --- |
| Time Period  (Early=dates before median index date)^^[[1]](#footnote-1)^^ | Early | Recent | Early | Recent | Early | Recent | Early | Recent |
| **Metropolitan Influence Zones** |  |  |  |  |  |  |  |  |
| Urban: POP 1, 500, 000+ | Ref | Ref | Ref | Ref | Ref | Ref | Ref | Ref |
| Urban: POP 500, 000-1,499, 999 | 1.04 (0.89, 1.21) | 1.10 (0.94, 1.28) | 1.07 (0.80, 1.44) | 1.24 (0.94, 1.66) | 0.74 (0.66, 0.82) | 0.81 (0.74, 0.91) | 0.90 (0.81, 1.00) | 0.93 (0.84, 1.02) |
| Urban: POP 100, 000-499, 999 | 1.17 (1.04, 1.32) | 1.22 (1.08, 1.38) | 0.85 (0.66, 1.08) | 1.03 (0.81, 1.30) | 0.96 (0.89, 1.04) | 0.87 (0.81, 0.94) | 1.00 (0.92, 1.08) | 1.08 (0.99, 1.16) |
| Urban: POP 10, 000-99, 999 | 1.34 (1.13, 1.59) | 1.58 (1.33, 1.87) | 0.79 (0.55, 1.14) | 1.42 (1.01, 1.99) | 1.32 (1.18, 1.47) | 1.19 (1.07, 1.34) | 1.24 (1.31, 1.13) | 1.28 (1.14, 1.43) |
| Rural: Strong MIZ | 1.63 (1.34, 1.99) | 1.93 (1.58, 2.35) | 1.37 (0.89, 2.11) | 1.40 (0.92, 2.14) | 1.03 (0.89, 1.20) | 1.03 (0.89, 1.19) | 1.31 (1.13, 1.53) | 1.09 (0.93, 1.26) |
| Rural: Moderate MIZ | 1.94 (1.59, 2.36) | 1.71 (1.39, 2.12) | 1.18 (0.74, 1.89) | 1.56 (0.99, 2.45) | 1.23 (1.06, 1.44) | 0.98 (0.84, 1.14) | 1.31 (1.13, 1.53) | 1.29 (1.10, 1.35) |
| Rural: Weak MIZ | 1.73 (1.29, 2.31) | 2.71 (2.02, 3.63) | 1.01 (0.51, 2.01) | 1.06 (0.55, 2.02) | 1.58 (1.28, 1.94) | 1.92 (1.56, 2.37) | 1.34 (1.08, 1.66) | 1.53 (1.23, 1.91) |
| **Rurality Index of Ontario** |  |  |  |  |  |  |  |  |
| Urban (Ref) | Ref | Ref | Ref | Ref | Ref | Ref | Ref | Ref |
| Small town | 1.33 (1.20, 1.48) | 1.39 (1.24, 1.55) | 0.96 (0.76, 1.21) | 1.23 (0.98, 1.54) | 1.19 (1.10, 1.28) | 1.11 (1.03, 1.20) | 1.16 (1.08, 1.26) | 1.09 (1.01, 1.18) |
| Rural | 1.70 (1.42, 2.03) | 1.70 (1.41, 2.04) | 0.92 (0.59, 1.42) | 1.24 (0.83, 1.85) | 1.31 (1.15, 1.50) | 1.43 (1.25, 1.64) | 1.29 (1.12, 1.49) | 1.24 (1.08, 1.42) |

eTable 6: Fully adjusted models with imputed missing variables

| Exposure | Suicide: Male | Suicide: Female | Suicide Attempt: Male | Suicide Attempt: Female |
| --- | --- | --- | --- | --- |
| **Metropolitan Influence Zones** |  |  |  |  |
| Urban: POP 1, 500, 000+ | Ref | Ref | Ref | Ref |
| Urban: POP 500, 000-1,499, 999 | 1.07 (0.96, 1.19) | 1.16 (0.94, 1.41) | 0.77 (0,72, 0.83) | 0.92 (0.85, 0.98) |
| Urban: POP 100, 000-499, 999 | 1.21 (1.10, 1.31) | 0.96 (0.81, 1.13) | 0.92 (0.87, 0.97) | 1.04 (0.98, 1.10) |
| Urban: POP 10, 000-99, 999 | 1.46 (1.29, 1.64) | 1.10 (0.86, 1.40) | 1.25 (1.16, 1.36) | 11.28 (1.18, 1.38) |
| Rural: Strong MIZ | 1.77 (1.54, 2.03) | 1.37 (1.02, 1.84) | 1.03 (0.93, 1.14) | 1.20 (1.08, 1.34) |
| Rural: Moderate MIZ | 1.81 (1.57, 2.09) | 1.31 (0.95, 1.80) | 1.12 (1.01, 1.24) | 1.32 (1.18, 1.47) |
| Rural: Weak MIZ | 2.24 (1.86, 2.70) | 2.19 (1.52, 3.14) | 2.10 (1.93, 2.48) | 2.54 (2.24, 2.88) |
| **Rurality Index of Ontario** |  |  |  |  |
| Urban | Ref | Ref | Ref | Ref |
| Small town | 1.33 (1.23, 1.44) | 1.06 (0.91, 1.24) | 1.12 (1.07, 1.18) | 1.09 (1.03, 1.15) |
| Urban | 1.67 (1.47, 1.90) | 1.03 (0.78, 1.37) | 1.33 (1.21, 1.46) | 1.23 (1.12, 1.36) |

eTable 7: Descriptive Statistics by Sex: Male Death by Suicide

| Variables | Case (N=7240)  N (%) | Control (N=28960)  N (%) | Standardized Difference |
| --- | --- | --- | --- |
| RIO Score |  |  |  |
| Mean (SD) | 12.92 (19.33) | 10.20 (16.85) | 0.150 |
| RIO Category |  |  |  |
| Urban | 552 (7.62) | 1495 (5.16) | 0.128 |
| Small Urban | 1863 (25.73) | 6464 (22.32) | 0.080 |
| Rural | 552 (7.62) | 1495 (5.16) | 0.101 |
| Age | 48.87 (16.55) | 48.87 (16.55) | <0.001 |
| Immigration status | 6660 (92.00) | 24147 (83.38) | 0.264 |
| non-immigrant | 421 (5.810 | 3919 (13.53) | 0.263 |
| Immigrant | 159 (2.20) | 894 (3.09) | 0.056 |
| Refugee |  |  |  |
| Neighbourhood Income Quintile |  |  |  |
| Q1 (lowest) | 1769 (24.64) | 5307 (18.40) | 0.149 |
| Q2 | 1551 (21.61) | 5701 (19.77) | 0.043 |
| Q3 | 1439 (20.05) | 5783 (20.05) | 0.002 |
| Q4 | 1262 (17.58) | 6052 (20.98) | 0.088 |
| Q5 (Highest) | 1157 (16.12) | 5997 (20.79) | 0.122 |
| Instability Index Quintile |  |  |  |
| 1 (lowest instability) | 991 (13.93) | 6033 (21.00) | 0.190 |
| 2 | 1161 (16.32) | 5659 (19.70) | 0.092 |
| 3 | 1353 (19.01) | 5359 (18.66) | 0.005 |
| 4 | 1502 (21.11) | 5427 (18.89) | 0.050 |
| 5 (highest instability) | 2109 (29.64) | 6246 (21.74) | 0.175 |
| Dependency Quintile |  |  |  |
| 1 (lowest dependency) | 1460 (20.52) | 6909 (24.05) | 0.089 |
| 2 | 1356 (19.14) | 5793 (20.17) | 0.030 |
| 3 | 1322 (18.58) | 5542 (19.29) | 0.023 |
| 4 | 1438 (20.21) | 5110 (17.79) | 0.057 |
| 5 (highest dependency) | 1534 (21.56) | 5370 (18.70) | 0.066 |
| Time Limited Minor (ADG1) | 1616 (22.32) | 5123 (17.69) | 0.116 |
| Time Limited Minor: Primary Infection (ADG2) | 2867 (39.60) | 10396 (35.90) | 0.076 |
| Time Limited Major (ADG3) | 979 (13.52) | 1503 (5.19) | 0.289 |
| Time Limited Major: Primary Infection (ADG4) | 1161 (16.04) | 2474 (8.54) | 0.223 |
| Allergies (ADG5) | 342 (4.62) | 1458 (5.03) | 0.014 |
| Asthma (ADG6) | 366 (5.06) | 1122 (3.87) | 0.057 |
| Likely to recur: Discrete (ADG7) | 2477 (34.21) | 7631 (26.35) | 0.172 |
| Likely to recur: Discrete infection (ADG8) | 1111 (15.35) | 3420 (11.81) | 0.103 |
| Likely to recur Progressive (ADG9) | 587 (8.11) | 812 (2.80) | 0.235 |
| Chronic Medical: Stable (ADG10) | 3261 (45.04) | 11493 (39.69) | 0.109 |
| Chronic Medical: Unstable (ADG11) | 2226 (30.75) | 5434 (18.76) | 0.280 |
| Chronic Specialty: Stable-Orthopedic (ADG12) | 241 (3.33) | 734 (2.53) | 0.047 |
| Chronic Specialty: Stable-Ear, Nose, Throat (ADG13) | 203 (2.80) | 631 (2.18) | 0.040 |
| Chronic Specialty-Stable-Eye (ADG14) | 398 (5.50) | 1409 (4.87) | 0.029 |
| Chronic Specialty: Unstable-Orthopedic (ADG16) | 277 (3.83) | 638 (2.20) | 0.095 |
| Chronic Specialty: Unstable-Ear, Nose, Throat (ADG17) | SUP | SUP | SUP |
| Chronic Specialty: Unstable-Eye (ADG18) | 1477 (5.10) | 399 (5.51) | 0.018 |
| Dermatologic (ADG20) | 778 (10.75) | 3405 (11.76) | 0.032 |
| Injuries/Adverse effects: Minor (ADG21) | 2274 (31.41) | 5359 (18.50) | 0.302 |
| Injuries/Adverse effects: Major (ADG22) | 2622 (36.22) | 4224 (14.59) | 0.513 |
| Psychosocial: Time Limited, Minor (ADG23) | 946 (13.07) | 959 (3.31) | 0.362 |
| Psychosocial: Recurrent or Persistent, Stable (ADG24) | 4289 (59.24) | 5547 (19.15) | 0.901 |
| Psychosocial: Recurrent or Persistent, Unstable (ADG25) | 2512 (34.70) | 1596 (5.51) | 0.782 |
| Signs/Symptoms: Minor (ADG26) | 2965 (40.95) | 8272 (28.56) | 0.262 |
| Signs/Symptoms: Uncertain (ADG27) | 4067 (56.17) | 11819 (40.81) | 0.311 |
| Signs/Symptoms: Major (ADG28) | 2334 (32.24) | 5704 (19.70) | 0.289 |
| Discretionary (ADG29) | 1311 (18.11) | 4424 (15.28) | 0.076 |
| See and Reassure (ADG30) | 173 (2.39) | 571 (1.97) | 0.039 |
| Prevention/Administrative (ADG31) | 2124 (29.34) | 8425 (29.09) | 0.005 |
| Malignancy (ADG32) | 700 (9.67) | 1955 (6.75) | 0.106 |
| Pregnancy (ADG33) | Male |  |  |
| Dental (ADG34) | 278 (3.84) | 481 (63.37) | 0.134 |
| Prior suicide attempt | 1076 (88.05) | 146 (11.95) | 0.560 |

eTable 8: Descriptive Statistics by Sex: Female Death by Suicide

| Variables | Case (N=2608) | Control (N=10432) | Standardized Difference |
| --- | --- | --- | --- |
| Rurality Index of Ontario Score |  |  |  |
| Mean (SD) | 9.92 (16.65) | 10.00 (16.68) | 0.868 |
| Rurality Index of Ontario Category |  |  |  |
| Urban | 1909 (73.20) | 7633 (73.17) | 0.001 |
| Small Urban | 565 (21.66) | 2254 (21.61) | 0.001 |
| Rural | 134 (5.14) | 545 (5.22) | 0.004 |
| Age | 48.36 (16.05) | 48.36 (16.04) | <0.001 |
| Immigration status |  |  |  |
| non-immigrant | 2357 (90.38) | 8680 (83.21) | 0.213 |
| Immigrant | 201 (7.71) | 1493 (14.31) | 0.212 |
| Refugee | 50 (1.92) | 259 (2.48) | 0.039 |
| Income Quintile |  |  |  |
| Q1 (lowest) | 754 (29.13) | 1915 (18.44) | 0.250 |
| Q2 | 541 (20.90) | 2015 (19.41) | 0.036 |
| Q3 | 459 (17.74) | 2115 (20.37) | 0.068 |
| Q4 | 436 (16.46) | 2166 (20.86) | 0.114 |
| Q5 (Highest) | 408 (15.77) | 2172 (20.92) | 0.134 |
| Instability Index Quintile |  |  |  |
| 1 | 302 (11.83) | 2203 (21.29) | 0.260 |
| 2 | 381 (14.93) | 1969 (19.03) | 0.114 |
| 3 | 416 (16.30) | 1944 (18.79) | 0.071 |
| 4 | 505 (19.79) | 1989 (19.22) | 0.008 |
| 5 | 948(37.15) | 2243 (21.68) | 0.332 |
| Dependency Quintile |  |  |  |
| 1 | 622 (24.37) | 2480 (23.97) | 0.002 |
| 2 | 471 (18.46) | 2099 (20.28) | 0.053 |
| 3 | 449 (17.59) | 1894 (18.30) | 0.025 |
| 4 | 427 (16.73) | 1812 (17.51) | 0.027 |
| 5 | 583 (22.84) | 2063 (19.94) | 0.063 |
| Time Limited Minor (ADG1) | 912 (34.97) | 2917 (27.96) | 0.151 |
| Time Limited Minor: Primary Infection (ADG2) | 1375 (52.72) | 4898 (46.95) | 0.116 |
| Time Limited Major (ADG3) | 497 (19.06) | 560 (5.37) | 0.428 |
| Time Limited Major : Primary Infection (ADG4) | 536 (20.55) | 872 (8.36) | 0.352 |
| Allergies (ADG5) | 201 (7.71) | 784 (7.52) | 0.007 |
| Asthma (ADG6) | 254 (9.74) | 615 (5.90) | 0.144 |
| Likely to recur: discrete (ADG7) | 1331 (51.04) | 3730 (35.76) | 0.312 |
| Likely to recur: discrete infection (ADG8) | 780 (29.91) | 2289 (21.94) | 0.183 |
| Likely to recur Progressive (ADG9) | 265 (10.16) | 205 (1.97) | 0.349 |
| Chronic Medical: Stable (ADG10) | 1374 (52.68) | 4372 (41.91) | 0.217 |
| Chronic Medical: Unstable (ADG11) | 968 (37.12) | 1820 (17.45) | 0.453 |
| Chronic Specialty: Stable-Orthopedic (ADG12) | 97 (3.72) | 257 (2.46) | 0.073 |
| Chronic Specialty: Stable-Ear, Nose, Throat (ADG13) | 64 (2.45) | 202 (1.94) | 0.035 |
| Chronic Specialty-Stable-Eye (ADG14) | 162 (6.21) | 549 (5.26) | 0.041 |
| Chronic Specialty: Unstable-Orthopedic (ADG16) | 141 (5.41) | 236 (2.26) | 0.164 |
| Chronic Specialty: Unstable-Ear, Nose, Throat (ADG17) | SUP | SUP | SUP |
| Chronic Specialty: Unstable-Eye (ADG18) | 180 (6.90) | 586 (5.62) | 0.053 |
| Dermatologic (ADG20) | 392 (15.03) | 1576 (15.11) | 0.002 |
| Injuries/Adverse effects: Minor (ADG21) | 1003 (38.46) | 2072 (19.86) | 0.418 |
| Injuries/Adverse effects: Major (ADG22) | 1282 (49.16) | 1441 (13.81) | 0.823 |
| Psychosocial: Time Limited, Minor (ADG23) | 530 (20.32) | 472 (4.52) | 0.493 |
| Psychosocial: Recurrent or Persistent, Stable (ADG24) | 2049 (78.57) | 2893 (27.73) | 1.180 |
| Psychosocial: Recurrent or Persistent, Unstable (ADG25) | 1243 (47.66) | 498 (4.77) | 1.120 |
| Signs/Symptoms: Minor (ADG26) | 1499 (57.48) | 3799 (36.42) | 0.432 |
| Signs/Symptoms: Uncertain (ADG27) | 1865 (71.51) | 5472 (52.45) | 0.400 |
| Signs/Symptoms: Major (ADG28) | 1416 (54.29) | 3752 (35.97) | 0.375 |
| Discretionary (ADG29) | 619 (23.73) | 1858 (17.81) | 0.146 |
| See and Reassure (ADG30) | 70 (2.68) | 231 (2.21) | 0.030 |
| Prevention/Administrative (ADG31) | 1203 (46.13) | 4725 (45.29) | 0.017 |
| Malignancy (ADG32) | 228 (8.74) | 718 (6.88) | 0.069 |
| Pregnancy (ADG33) | 110 (4.22) | 596 (5.71) | 0.069 |
| Dental (ADG34) | 108 (4.14) | 173 (1.66) | 0.148 |
| Prior suicide attempt | 756 (91.30) | 1852 (15.17) | 0.868 |

eTable 9: Descriptive Statistics by Sex: Male Suicide Attempt

| Variables | Case (N=39446)  N (%) | Control (N=78892)  N (%) | Standardized Difference |
| --- | --- | --- | --- |
| RIO Score |  |  |  |
| Mean (SD) | 11.05 (17.89) | 9.96 (16.54) | 0.063 |
| RIO Category |  |  |  |
| Urban | 27910 (70.75) | 57667 (73.10) | 0.052 |
| Small Urban | 9207 (23.34) | 17335 (21.97) | 0.033 |
| Rural | 2329 (5.90) | 3890 (4.93) | 0.043 |
| Age | 39.83 (15.81) | 39.84 (15.81) | 0.011 |
| Sex (% female) |  |  |  |
| Immigration status |  |  |  |
| non-immigrant | 36017 (91.31) | 65487 (83.01) | 0.250 |
| Immigrant | 2498 (6.33) | 10859 (13.76) | 0.249 |
| Refugee | 931 (2.36) | 2546 (3.23) | 0.053 |
| Neighbourhood Income Quintile |  |  |  |
| Q1 (lowest) | 11468 (29.34) | 14904 (18.97) | 0.240 |
| Q2 | 8458 (21.64) | 15800 (20.11) | 0.035 |
| Q3 | 7151 (18.29) | 15856 (20.17) | 0.050 |
| Q4 | 6440 (16.48) | 16555 (21.07) | 0.120 |
| Q5 (Highest) | 5571 (14.25) | 15471 (19.69) | 0.147 |
| Instability Index Quintile |  |  |  |
| 1 (lowest instability) | 4933 (12.80) | 16851 (21.53) | 0.238 |
| 2 | 5635 (14.62) | 15364 (19.63) | 0.139 |
| 3 | 6633 (17.21) | 14367 (18.36) | 0.037 |
| 4 | 8387 (21.76) | 14697 (18.78) | 0.066 |
| 5 (highest instability) | 12953 (33.61) | 16976 (21.69) | 0.237 |
| Dependency Quintile |  |  |  |
| 1 (lowest dependency) | 8867 (22.49) | 20069 (25.65) | 0.082 |
| 2 | 7779 (20.18) | 16551 (21.15) | 0.031 |
| 3 | 7357 (19.09) | 14823 (18.94) | 0.004 |
| 4 | 7218 (18.73) | 13719 (17.53) | 0.024 |
| 5 (highest dependency) | 7520 (19.51) | 13093 (16.73) | 0.065 |
| Time Limited Minor (ADG1) | 10796 (27.37) | 13301 (16.86) | 0.255 |
| Time Limited Minor: Primary Infection (ADG2) | 18729 (47.48) | 28523 (36.15) | 0.231 |
| Time Limited Major (ADG3) | 4153 (10.53) | 2811 (3.56) | 0.275 |
| Time Limited Major: Primary Infection (ADG4) | 7331 (18.58) | 5928 (7.51) | 0.333 |
| Allergies (ADG5) | 2175 (5.51) | 4159 (5.27) | 0.011 |
| Asthma (ADG6) | 2658 (6.74) | 3066 (3.89) | 0.127 |
| Likely to recur: Discrete (ADG7) | 14544 (36.87) | 17690 (22.42) | 0.320 |
| Likely to recur: Discrete infection (ADG8) | 6033 (15.29) | 8413 (10.66) | 0.138 |
| Likely to recur Progressive (ADG9) | 2384 (6.04) | 1336 (1.69) | 0.227 |
| Chronic Medical: Stable (ADG10) | 15019 (38.07) | 21863 (27.71) | 0.222 |
| Chronic Medical: Unstable (ADG11) | 8677 (24.53) | 9754 (12.36) | 0.318 |
| Chronic Specialty: Stable-Orthopedic (ADG12) | 1337 (3.39) | 1740 (2.21) | 0.072 |
| Chronic Specialty: Stable-Ear, Nose, Throat (ADG13) | 709 (1.80) | 1246 (1.58) | 0.017 |
| Chronic Specialty-Stable-Eye (ADG14) | 1464 (3.71) | 2066 (2.62) | 0.062 |
| Chronic Specialty: Unstable-Orthopedic (ADG16) | 1673 (4.24) | 1246 (1.58) | 0.159 |
| Chronic Specialty: Unstable-Ear, Nose, Throat (ADG17) | SUP | SUP | SUP |
| Chronic Specialty: Unstable-Eye (ADG18) | 1513 (3.84) | 2647 (3.36) | 0.026 |
| Dermatologic (ADG20) | 4345 (11.02) | 8649 (10.96) | 0.002 |
| Injuries/Adverse effects: Minor (ADG21) | 15526 (39.36) | 14642 (18.56) | 0.471 |
| Injuries/Adverse effects: Major (ADG22) | 27547 (69.83) | 11118 (14.09) | 1.369 |
| Psychosocial: Time Limited, Minor (ADG23) | 6805 (17.25) | 2257 (2.86) | 0.493 |
| Psychosocial: Recurrent or Persistent, Stable (ADG24) | 27513 (69.75) | 14074 (17.84) | 1.228 |
| Psychosocial: Recurrent or Persistent, Unstable (ADG25) | 18194 (46.12) | 3480 (4.41) | 1.094 |
| Signs/Symptoms: Minor (ADG26) | 16975 (43.04) | 19212 (24.35) | 0.403 |
| Signs/Symptoms: Uncertain (ADG27) | 24236 (61.44) | 28362 (35.95) | 0.527 |
| Signs/Symptoms: Major (ADG28) | 12531 (31.77) | 11897 (15.08) | 0.402 |
| Discretionary (ADG29) | 7471 (18.94) | 10327 (13.09) | 0.160 |
| See and Reassure (ADG30) | 627 (1.59) | 952 (1.21) | 0.033 |
| Prevention/Administrative (ADG31) | 10285 (26.07) | 18824 (23.86) | 0.051 |
| Malignancy (ADG32) | 2143 (5.43) | 2933 (3.72) | 0.082 |
| Pregnancy (ADG33) | 74 (0.19) | 66 (0.08) | 0.028 |
| Dental (ADG34) | 2370 (6.01) | 1294 (1.64) | 0.229 |

eTable 10: Descriptive Statistics by Sex: Female Suicide Attempt

| Variables | Case (N=43034) | Control (N=86068) | Standardized Difference |
| --- | --- | --- | --- |
| Rurality Index of Ontario Score |  |  |  |
| Mean (SD) | 10.89 (17.67) | 9.70 (16.30) | 0.070 |
| Rurality Index of Ontario Category |  |  |  |
| Urban | 30696 (71.33) | 63435 (73.70) | 0.053 |
| Small Urban | 9847 (22.88) | 18548 (21.55) | 0.032 |
| Rural | 2491 (5.79) | 4085 (4.75) | 0.047 |
| Age | 40.08 (16.31) | 40.08 (16.29) | <0.001 |
| sex (% female) |  |  |  |
| Immigration status |  |  |  |
| non-immigrant | 38901 (90.40) | 71184 (82.71) | 0.227 |
| Immigrant | 3249 (7.55) | 12658 (14.71) | 0.229 |
| Refugee | 884 (2.05) | 2226 (2.59) | 0.035 |
| Income Quintile |  |  |  |
| Q1 (lowest) | 12197 (28.58) | 16189 (18.88) | 0.226 |
| Q2 | 9154 (21.45) | 16696 (19.48) | 0.047 |
| Q3 | 7728 (18.11) | 1783 (20.16) | 0.054 |
| Q4 | 7125 (16.70) | 181107 (21.12) | 0.115 |
| Q5 (Highest) | 6473 (15.17) | 17452 (20.36) | 0.138 |
| Instability Index Quintile |  |  |  |
| 1 | 5887 (13.99) | 18842 (22.06) | 0.216 |
| 2 | 6287 (14.94) | 16513 (19.33) | 0.122 |
| 3 | 7089 (16.85) | 15527 (18.18) | 0.042 |
| 4 | 9184 (21.83) | 16185 (18.95) | 0.063 |
| 5 | 13623 (32.38) | 18347 (21.48) | 0.236 |
| Dependency Quintile |  |  |  |
| 1 | 10112 (24.04) | 22406 (26.23) | 0.059 |
| 2 | 8424 (20.02) | 17884 (20.94) | 0.030 |
| 3 | 7760 (18.45) | 15920 (18.64) | 0.012 |
| 4 | 7473 (17.76) | 14670 (17.18) | 0.009 |
| 5 | 8301 (19.73) | 14534 (17.02) | 0.063 |
| Time Limited Minor (ADG1) | 15904 (36.96) | 22534 (26.18) | 0.233 |
| Time Limited Minor: Primary Infection (ADG2) | 26571 (61.74) | 42154 (48.98) | 0.259 |
| Time Limited Major (ADG3) | 4742 (11.02) | 3991 (4.64) | 0.239 |
| Time Limited Major : Primary Infection (ADG4) | 7323 (17.02) | 7105 (8.26) | 0.266 |
| Allergies (ADG5) | 3719 (8.64) | 6366 (7.40) | 0.046 |
| Asthma (ADG6) | 4583 (10.65) | 4799 (5.58) | 0.187 |
| Likely to recur: discrete (ADG7) | 21698 (50.42) | 30263 (35.16) | 0.312 |
| Likely to recur: discrete infection (ADG8) | 14814 (34.42) | 19850 (23.06) | 0.253 |
| Likely to recur Progressive (ADG9) | 2154 (5.01) | 1178 (1.37) | 0.208 |
| Chronic Medical: Stable (ADG10) | 18815 (43.72) | 28144 (32.70) | 0.228 |
| Chronic Medical: Unstable (ADG11) | 10831 (25.17) | 11907 (13.83) | 0.289 |
| Chronic Specialty: Stable-Orthopedic (ADG12) | 1485 (3.45) | 1734 (2.01) | 0.088 |
| Chronic Specialty: Stable-Ear, Nose, Throat (ADG13) | 896 (2.08) | 1305 (1.52) | 0.043 |
| Chronic Specialty-Stable-Eye (ADG14) | 1951 (4.53) | 3041 (3.53) | 0.051 |
| Chronic Specialty: Unstable-Orthopedic (ADG16) | 1682 (3.91) | 1420 (1.65) | 0.138 |
| Chronic Specialty: Unstable-Ear, Nose, Throat (ADG17) | SUP | SUP | SUP |
| Chronic Specialty: Unstable-Eye (ADG18) | 2034 (4.73) | 3683 (4.28) | 0.022 |
| Dermatologic (ADG20) | 6374 (14.81) | 13439 (15.61) | 0.022 |
| Injuries/Adverse effects: Minor (ADG21) | 15764 (36.63) | 16597 (19.28) | 0.394 |
| Injuries/Adverse effects: Major (ADG22) | 30381 (70.60) | 11304 (13.12) | 1.433 |
| Psychosocial: Time Limited, Minor (ADG23) | 8800 (20.45) | 3716 (4.32) | 0.505 |
| Psychosocial: Recurrent or Persistent, Stable (ADG24) | 34886 (81.07) | 22935 (26.65) | 1.303 |
| Psychosocial: Recurrent or Persistent, Unstable (ADG25) | 18242 (42.39) | 3221 (3.74) | 1.033 |
| Signs/Symptoms: Minor (ADG26) | 23881 (55.49) | 30598 (35.55) | 0.409 |
| Signs/Symptoms: Uncertain (ADG27) | 30008 (69.73) | 42653 (49.56) | 0.4202 |
| Signs/Symptoms: Major (ADG28) | 21866 (50.81) | 30406 (35.33) | 0.317 |
| Discretionary (ADG29) | 9293 (21.59) | 13510 (15.70) | 0.152 |
| See and Reassure (ADG30) | 1065 (2.47) | 1543 (1.79) | 0.047 |
| Prevention/Administrative (ADG31) | 20946 (48.67) | 42360 (49.22) | 0.011 |
| Malignancy (ADG32) | 2651 (6.16) | 4257 (4.95) | 0.053 |
| Pregnancy (ADG33) | 3303 (7.68) | 7532 (8.75) | 0.039 |
| Dental (ADG34) | 2209 (5.13) | 1484 (1.72) | 0.188 |

1. This analysis dichotomizes cases and controls based on the event/index date occurring prior to or following the median event date. [↑](#footnote-ref-1)
